# Cancer Drug Discovery and Development: Stem Cells and Cancer

**DOI:** 10.1038/sj.bjc.6605525

**Published:** 2010-02-16

**Authors:** M H Stewart

**Affiliations:** 1 MRC Molecular Haematology Unit, Weatherall Institute of Molecular Medicine, University of Oxford, Oxford, UK

The cancer stem cell (CSC) hypothesis is now widely accepted by basic cancer researchers and the implications of this theory for the development of new cancer therapies are beginning to be recognised. In this regard, *Stem Cells and Cancer* is a timely compilation that is targeted at cancer and stem cell researchers, clinicians and graduate students with the intention of providing a comprehensive overview of the state of the field. In addition to describing the current state of CSC research, *Stem Cells and Cancer* discusses the hurdles that need to be overcome before basic CSC research can be effectively translated into clinical therapies.

*Stem Cells and Cancer* begins by introducing the reader to the now widely accepted CSC hypothesis, which states that cancers arise hierarchically from relatively rare, treatment-resistant CSCs. This is followed by four general sections: *The Stem Cell Niche*; *Molecular Pathways and Gene Expression* (implicated in Stem Cells and Cancer); *Cancer Stem Cells in Solid Tumors*; and *Targeting Cancer Stem Cells with Therapy*, which are further divided into chapters that provide a more in-depth discussion of specific topics within each section. *Molecular Pathways and Gene Expression*, for example, has the unenviable mission of covering the important signalling molecules and pathways implicated in the regulation of both stem cells and CSCs. This is a complex topic and sometimes the authors fall into the trap of providing too much detail, but in general this section provides a good resource that compares and contrasts genes and pathways involved in stem and CSC regulation. The subsequent section, *Cancer Stem Cells in Solid Tumors*, does a solid job of providing the research history of a variety of tumour types, including mammary, glioma, gastrointestinal, hepatocellular carcinoma and prostate cancer, before moving onto recent advances. This section also addresses the current limitations within this field, specifically the phenotypic identification of CSCs within tumours and the functional assays, both *in vitro* and *in vivo*, required to show their presence. Two particularly interesting and well-written chapters, within this and the following section, *Targeting Cancer Stem Cells with Therapy*, address the role of angiogenesis and neurogenesis in tumour progression and, separately, provide a comprehensive discussion regarding the putative link between CSCs and tumour metastasis.

On a less positive note, there are some major flaws within this text. Some of the chapters alternate between providing a superficial overview and going into minute detail about some very specific aspect of the cell, tissue or pathway being discussed. A better balance is achieved in many chapters; however, often poor writing and composition sour the overall impression of the text. Sometimes, it seems that the authors are not entirely sure what point they are trying to make. This is shown by the occasionally inappropriate choices in chapter titles. Chapter 8, for example, is entitled ‘*Plasticity Underlying Multipotent Tumor Stem Cells*’, but would have been more appropriated titled ‘The Role of Nodal signalling in Tumorogenesis’, which is the primary focus of this chapter. In addition, the lack of discussion surrounding the recent results from the study by Quintana *et al*, wherein it is strongly suggested that CSCs may not be such a rare event within tumours, is a major oversight in a text aiming to provide a comprehensive overview of CSCs. Space was obviously not a constraining factor, as three chapters are specifically and slightly excessively dedicated to prostate cancer and stem cells. Finally, the choice to use black and white figures within chapters and then placing a corresponding colour panel in the centre of the book creates a disjointed and off-putting feel to the text, especially as the black and white versions were often confusing because of lack of appropriately contrasting shades.

Overall, *Stem Cells and Cancer* is timely in its subject matter and is relatively successful at delivering a comprehensive and stimulating discussion of the current progress and roadblocks within CSC research. *Stem Cells and Cancer* contains many informative and interesting chapters, which are unfortunately tempered by occasional sections that are disjointed and lack focus. In general, this text provides a mostly solid base from which to pursue further investigation into this exciting and rapidly emerging field.

